# Characterization of ferroptosis signature to evaluate the predict prognosis and immunotherapy in glioblastoma

**DOI:** 10.18632/aging.203257

**Published:** 2021-07-09

**Authors:** Xiaopeng Zhu, Yuxiang Zhou, Yangqian Ou, Zebo Cheng, Deqing Han, Zhou Chu, Sian Pan

**Affiliations:** 1 Department of Neurosurgery, Zhuzhou Central Hospital, Zhuzhou 412000, Hunan Province, PR China; 2 Department of Operating Theatre, Zhuzhou Central Hospital, Zhuzhou 412000, Hunan Province, PR China; 3 Department of Rehabilitation Medicine, Zhuzhou Central Hospital, Zhuzhou 412000, Hunan Province, PR China

**Keywords:** ferroptosis, gene signature, glioblastoma prognosis, immune, personalized therapy

## Abstract

Background: Glioblastoma (GBM) is the most common type of brain cancer with poor survival outcomes and unsatisfactory response to current therapeutic strategies. Recent studies have demonstrated that ferroptosis-related genes (FRGs) are linked with the occurrence and development of GBM and may become promising biological indicators in GBM therapy.

Methods: We systematically assessed the relationship between FRGs expression profiles and prognosis in glioma patients based on the Cancer Genome Atlas (TCGA) and Chinese Glioma Genome Atlas (CGGA) datasets to establish a risk score model according to the gene signature of multiple survival-associated DEGs. Further, the differences between the tumor microenvironment score, immune cell infiltration, immune checkpoint expression levels, and drug sensitivity in the high- and low-risk group are analyzed through a variety of algorithms in R software.

Results: GBM patients were divided into two subgroups (high- and low-risk) according to the established risk score model. Patients in the high-risk group showed significantly reduced overall survival compared with those in the low-risk group. Also, we found that the high-risk group showed higher ImmuneScore and StromalScore, while different subgroups have significant differences in immune cell infiltration, immune checkpoint expression levels, and drug sensitivity. In summary, we developed and validated an FRGs risk model, which served as an independent prognostic indicator for GBM. Besides, the two subgroups divided by the model have significant differences, which provides novel insights for further studies as well as the personalized treatment of patients.

## INTRODUCTION

Clinically, glioblastoma (GBM) ranks as most prevalent central nervous system cancer comprising 55.4% of all and 15% of all the central nervous systems, with an occurring percentage of 3.2% out of 100,000 people [1–4]. World Health Organization (WHO) has classified glioblastoma as the grade IV of astrocytic tumors, characterized by mitotic activity, anaplasia, cytological atypia, microvascular proliferation, and necrosis [5–7]. In recent decades, although there are a variety of therapeutic strategies including surgery, radiotherapy, and chemotherapy, it remains incurable with recurrence and poor prognosis, and infected patients after diagnosis survive for 15 months with a 5.5% 5-year survival rate [8–10]. Meanwhile, the increasing incidence of glioma raises the importance and urgency of its diagnosis and therapies. To further improve the survival period and quality of life of patients, accurate molecular biomarkers are urgently needed.

Recently, ferroptosis is an emerging approach that has fetched much attention from researchers. It is considered an iron-dependent cell death, which is triggered by high levels of lipid hydroperoxide [11–14]. Ferroptosis contribute to occurrence and development of diverse disorders e.g., blood diseases, kidney damage, nervous system diseases, and cancers [15–17]. For instance, non-thermal plasma (NTP) splits ferritin and reduces Fe_3_^+^ to Fe_2_^+^, eliminating oral squamous cell carcinoma cells [18]. In lung cancer, blockade of NFS1, induces iron-starvation response and ferroptosis [19]. Besides, withaferin A can kill tumors in high-risk neuroblastoma via Kelch-like ECH-associated protein 1 (KEAP1) -Nrf2 axis, a noncanonical ferroptosis pathway [20]. Furthermore, it is reported that cisplatin together with inhibition of GPX4 can initiate ferroptosis and synergistically improved chemotherapeutic efficacy in GBM [21]. However, the effect of ferroptosis and ferroptosis-related genes in GBM is still not well studied, and they are great treatment value in GBM that is worthy of further researches.

In the current investigation, the features of ferroptosis-related genes (FRGs) in GBM were characterized using data from the TCGA and CGGA data sets. An individualized signature of FRGs was constructed and validated for GBM patients, which holds promising prospects for diagnosis and prognosis application in the future. In addition, the FRGs model classified GBM patients into two subgroups. Different subgroups have significant differences in tumor microenvironment score, immune cell infiltration, and immune checkpoint expression levels, which may provide help for the development of GBM novel immunotherapy. Also, we investigated candidate drugs targeting this FRGs-related signature via the publicly available drug sensitivity database. At last, we preliminarily performed the *in vitro* protein expression level of the genes in our model through western blot.

## MATERIALS AND METHODS

### Data collection

RNA sequencing data (TCGA-GBM) of 169 GBM samples and 5 normal tissue samples with their clinical details were retrieved from TCGA (http://cancergenome.nih.gov/). However, some samples were excluded which were without survival time and finally 165 GBM samples from TCGA were considered in this study (Supplementary Table 1). Using DESeq2 package, we carried out preprocessing of raw data. Subsequently, the |log2 fold change (FC)| ≥ 1 and false discovery rate (FDR) < 0.05 were employed to identify differentially expressed FRGs. The clinical information and RNA-seq transcriptome data of the samples (dataset ID: mRNAseq_325, dataset ID: mRNAseq_693) were retrieved from the CGGA (http://www.cgga.org.cn). In this study, TCGA data was used as the training set, and the CGGA data was used as the validation set. Next, 60 genes involved in ferroptosis were obtained from published studies [22–25] which are outlined in Supplementary Table 2.

### Design of a PPI network

With the aim of analyzing the protein-protein interaction information between different genes, the STRING (https://string-db.org/) online platform was utilized to evaluate the interactions among those FRGs and hided disconnected nodes in the network. The Cytoscape_v3.7.0 software was applied in the construction of a visual PPI network [26–29].

### KEGG pathway and GO enrichment analysis

The “clusterProfiler” R package [30] was applied in the enrichment analysis of differentially expressed FRGs based on Kyoto Encyclopedia of Genes and Genomes (KEGG) and Gene Ontology (GO) and analyses. *P* < 0.05 represented statistical significance.

### Prognostic model construction and evaluation

Based on the FRGs in TCGA first selected prognosis-related FRGs by univariate Cox regression, a multivariate Cox proportional hazards regression model was designed for predicting GBM patients’ prognosis. In this model, we determined risk scores for each sample according to the following formula:

$$Risk\;Score=\sum_{i=1}^{n}Expi\;\beta i$$

Where Exp is the gene expression value, β stands for the regression coefficient [31–33].

Further, the GBM patients were assigned into low- and high-risk groups based on risk score cutoff values. The groups were subjected to survival analysis using the Kaplan-Meier method with the log-rank test. In addition, an ROC curve was developed using the “SurvivalROC” R package which was then applied in evaluation of the prognosis prediction ability of the designed model. A nomogram was drawn to assess the OS based on the “rms” R package. The performance of the nomogram was further clarified using CGGA as the validation cohort. *P* < 0.05 represented statistical significance.

### Generation of ImmuneScore and StromalScore

The ratio of immune to stromal components for every sample in the tumor microenvironment was determined using the ESTIMATE package [34], which was shown as two scores: StromalScore and ImmuneScore. Higher scores correlated with higher ratios of in the tumor microenvironment.

### Calculation of immune cell type fractions

The CIBERSORT was applied for estimating 22 immune cell types fractions (neutrophils, eosinophils, activated mast cells, resting mast cells, activated dendritic cells, resting dendritic cells, macrophages M2, macrophages M1, macrophages M0, monocytes, activated NK cells, resting NK cells, gamma delta T cells, T cells regulatory, follicular helper T cells, activated memory CD4 T cells, resting memory CD4 T cells, naive CD4 T cells, CD8 T cells, plasma cells, memory B cells, and naive B cells) between subjected with high -and low-risk scores.

### GSEA functional analysis

To characterize pathways associated with low- and high-risk subgroups, the Gene Set Enrichment Analysis (GSEA software, version 4.0.1) was applied. We set number of random sample permutations at 1000, and significance cut-off values at *P* < 0.05.

### Cell culture

The normal human glial cell line (HEB) was bought from Otwo Biotech (China). HS 683 cell line, H4 cell line, and U251 cell line were all purchased from the Cell Resource Center, Peking Union Medical College. These cells were passaged with DMEM (Hyclone), enriched with 10% FBS (Hyclone) in 37 Celsius and 5% CO2 incubator.

### Western blot

The protein expression levels were determined as previously reported with some modifications [35–37]. Antibodies used were: Anti-STEAP3 (1/1000 dilution, Abcam, ab151566, UK), Anti-CRYAB (1/1000 dilution, Abcam, ab76467, UK), Anti-MT1G (1/1000 dilution, Omnimab, #OM263051, USA).

### Statistical analysis

The “survminer” package in R was applied in univariate and multivariate Cox regression analyses. The ROC curve and AUC were obtained using the “timeROC” package in R. The “ggplotify” “VennDiagram,” “maftools,” “plot3D,” “cowplot,” “ggforest,” and “ggplot2,” packages in R were used for visualization. The results were expressed as mean ± SEM. Statistical analysis was done using SPSS.22 and R software version 4.0.3. *P* < 0.05 was chosen as the threshold of statistically significance.

### Ethics approval and consent to participate

Animal and human experiments were not conducted in this study.

### Availability of data and material

Technical appendix, statistical code, and dataset are available from the corresponding author at [090102080@m.fafu.edu.cn].

## RESULTS

### Determination of differently expressed FRGs in GBM patients

**Figure 1:**
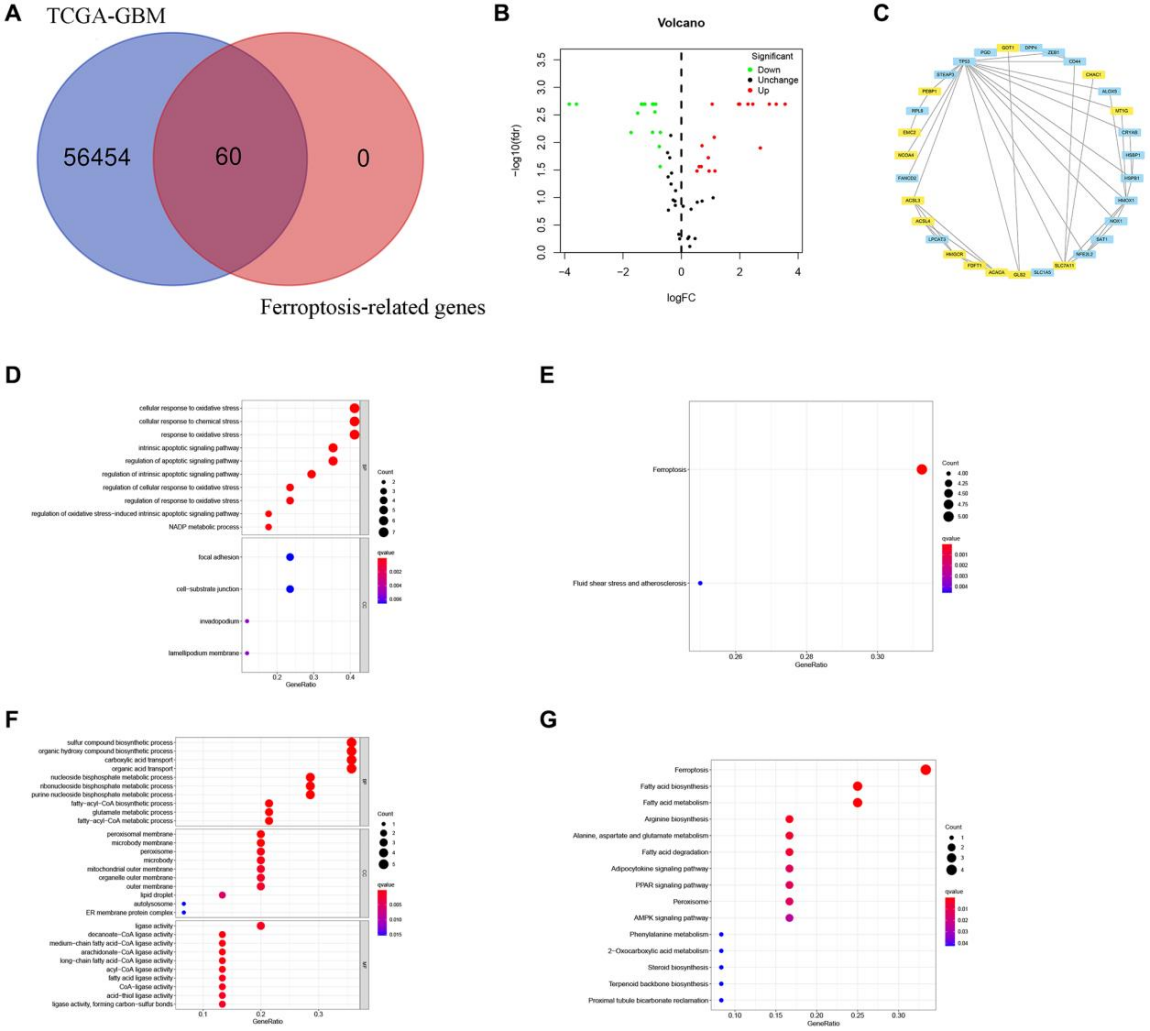
**Identification of FRGs in GBM.** (**A**) A Venn diagram indicating that 60 FRGs were identified in the TCGA-GBM cohorts. (**B**) Volcano plot showing DEGs among FRGs in GBM. (**C**) A PPI network on the relationship between up-regulated and down-regulated DEGs. Blue or yellow are up-or down-regulated DEGs, respectively. (**D**) GO analysis of up-regulated DEGs. (**E**) KEGG analysis of up-regulated DEGs. (**F**) GO analysis of down-regulated DEGs. (**G**) KEGG analysis of down-regulated DEGs.

Firstly, we extracted FRGs from the TCGA-GBM as presented in (Figure 1A). The differential gene expression of FRGs between the two groups identified 17 upregulated and 15 downregulated FRGs (Figure 1B). The STRING and Cytoscape were used to visualize the interactions among FRGs. Moreover, FRGs disconnected nodes in the network were not shown (Figure 1C).

### Results of GO and KEGG analysis

Based on GO analysis, upregulated differently expressed FRGs were strongly linked to cellular response to oxidative stress, NADP metabolic process, regulation of oxidative stress−induced intrinsic apoptotic signaling pathway, cellular response to oxidative stress, response to oxidative stress, intrinsic apoptotic signaling pathway, apoptotic signaling pathway, cellular response to chemical stress, cell−substrate junction, focal adhesion, invadopodium, lamellipodium membrane, and intrinsic apoptotic signaling pathway (Figure 1D).

The GO results exhibited that downregulated differently expressed FRGs showed strongly enrichment in sulfur compound biosynthetic process, organic acid transport, carboxylic acid transport, several metabolic processes including purine nucleoside bisphosphate, fatty−acyl−CoA, ribonucleoside bisphosphate, nucleoside bisphosphate, and glutamate, fatty−acyl−CoA biosynthesis, organic hydroxy compound biosysnthesis, ER membrane protein complex, autolysosome, lipid droplet, microbody, peroxisome, peroxisomal membrane, medium−chain fatty acid−CoA ligase activity, arachidonate−CoA ligase activity, long−chain fatty acid−CoA ligase activity, and decanoate−CoA ligase activity (Figure 1F).

Moreover, the upregulated differently expressed FRGs were mainly enriched in Ferroptosis, Fluid shear stress, and atherosclerosis (Figure 1E), while downregulated FRGs were significantly enriched for Ferroptosis, Proximal tubule bicarbonate reclamation, Terpenoid backbone biosynthesis, Steroid biosynthesis, 2−Oxocarboxylic acid metabolism, Phenylalanine metabolism, AMPK signaling pathway, Peroxisome, PPAR signaling pathway, Adipocytokine signaling pathway, Fatty acid degradation, Alanine, aspartate and glutamate metabolism, Arginine biosynthesis, Fatty acid metabolism, Fatty acid biosynthesis (Figure 1G).

### Prognosis-related FRGs selection

**Figure 2:**
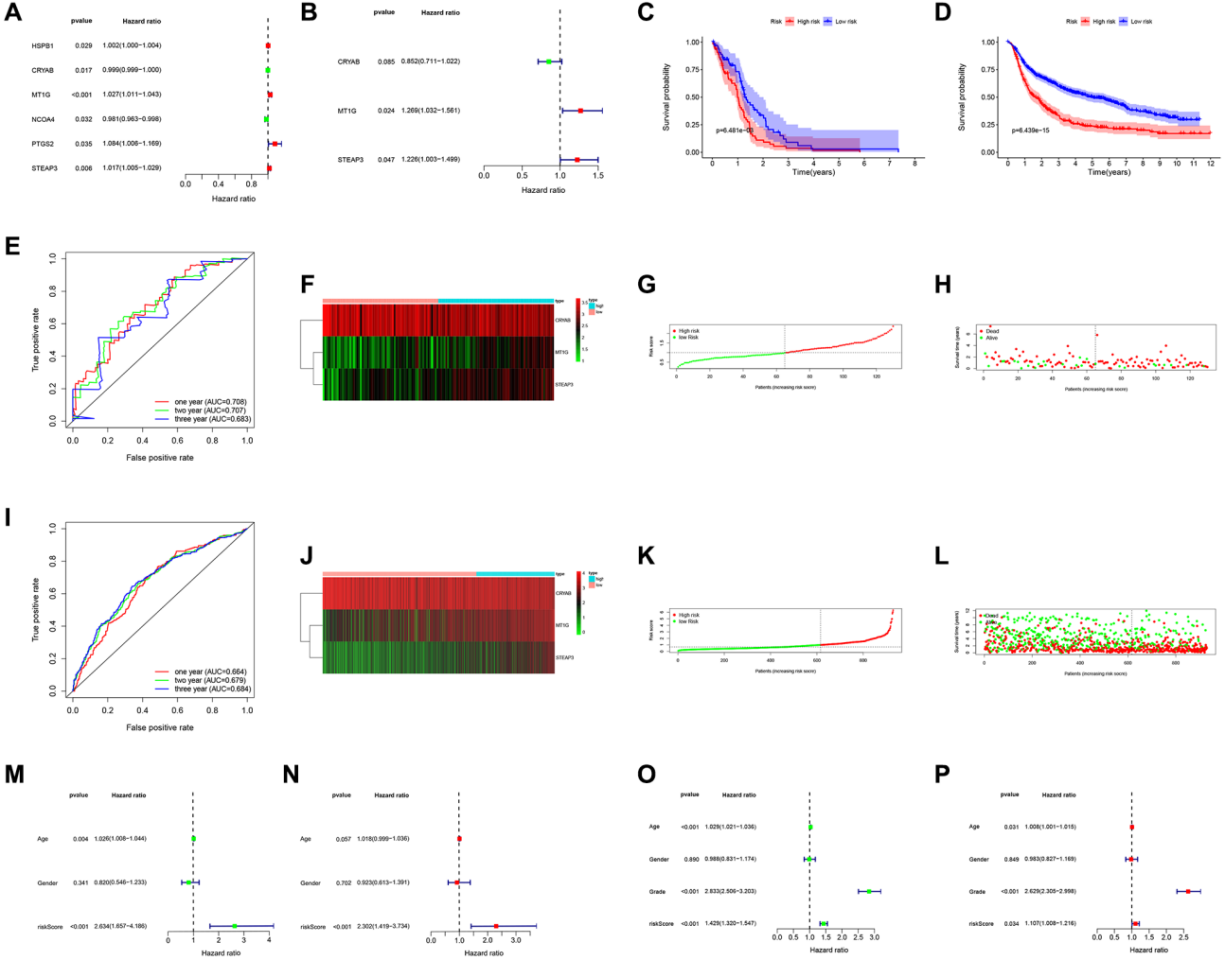
**Prognostic significance of the FRGs signature derived risk scores.** (**A**, **B**) Univariate and multivariate Cox analysis evaluating the prognostic-related genes in the TCGA (**A**) and CGGA cohort (**B**). The Kaplan-Meier survival curves for the high- and low-risk groups in TCGA (**C**) and CGGA cohort (**D**). (**E**, **I**) The predictive efficiency of the FRGs risk signature on the 1-, 3-, and 5-years survival rate in TCGA (**E**) and CGGA cohort (**I**) via ROC curve. (**F**, **J**) Heat maps of these three FRGs (CRYAB, MT1G, STEAP3) expression profiles in TCGA (**F**) and CGGA cohort (**J**). (**G**–**I**, **K**–**L**) Distribution of risk score and patient survival time, and status of GBM in TCGA (**G**, **H**) and CGGA cohort (**K**, **L**). The black dotted line is the optimal cut-off value for dividing patients into low-risk and high-risk groups. (**M**–**P**) Univariate and multivariate Cox analyses for evaluating the independent prognostic value of the FRGs signature in TCGA (**M**, **N**) and CGGA cohort (**O**, **P**).

A total of 6 prognostic-related hub FRGs were found (Figure 2A). Three of the six hub FRGs could independently predict the outcomes of GBM patients (Figure 2B, Table 1).

**Table 1:** Univariate and multivariate Cox regression analysis.

**Gene**	**Univariate analysis**		**Multivariate analysis**	
	**HR (95% CI)**	***p***	**HR (95% CI)**	***p***
HSPB1	1.002 (1.000–1.004)	0.029		
CRYAB	0.999 (0.999–1.000)	0.017	0.852 (0.711–1.022)	0.085
MT1G	1.027 (1.011–1.043)	0.000	1.269 (1.032–1.561)	0.024
NCOA4	0.981 (0.963–0.998)	0.032		
PTGS2	1.084 (1.005–1.169)	0.035		
STEAP3	1.017 (1.005–1.029)	0.006	1.226 (1.003–1.499)	0.047

### Construction and validation of prognosis risk score model

A prognosis prediction model was designed from the 3 hub FRGs. Using the model, a risk score for a given patient could be determined as:

$$Risk\;Score=\sum_{i=1}^{n}Expi\;\beta i$$

The model assigned the TCGA-GBM patients into low- and high-risk subgroups. The findings revealed that those in high-risk subgroup had worse OS relative to those in low-risk subgroup as displayed in Figure 2C. Analysis of a time-dependent ROC and AUC) of the FRGs model found a prognostic ability of 0.708 (one-year), 0.707 (two-year), and 0.683 (three-year), indicating a good performance (Figure 2E). Figure 2F–2H displays the heat map, risk score, and survival status of subjects in the 3 FRGs in both subgroups. We applied the formula to validate the prognostic significance of the model in other GBM patient cohorts using CGGA dataset as validation. Similarly, those with high-risk scores showed poorer OS than subjects with low-risk scores in the CGGA dataset (Figure 2D). Figure 2J–2L shows the survival status, risk score, and expression heat map of CGGA cohorts both risk groups, the FRGs signature built in this study has stable prognostic ability and a similar tendency of gene expression in both groups. The results presented in Figure 2M–2P depicted that risk score can could independently assess the progress of GBM patients. Hence the model had satisfactory specificity and sensitivity.

### Design of a nomogram and drug relevance using 3 hub FRGs

**Figure 3:**
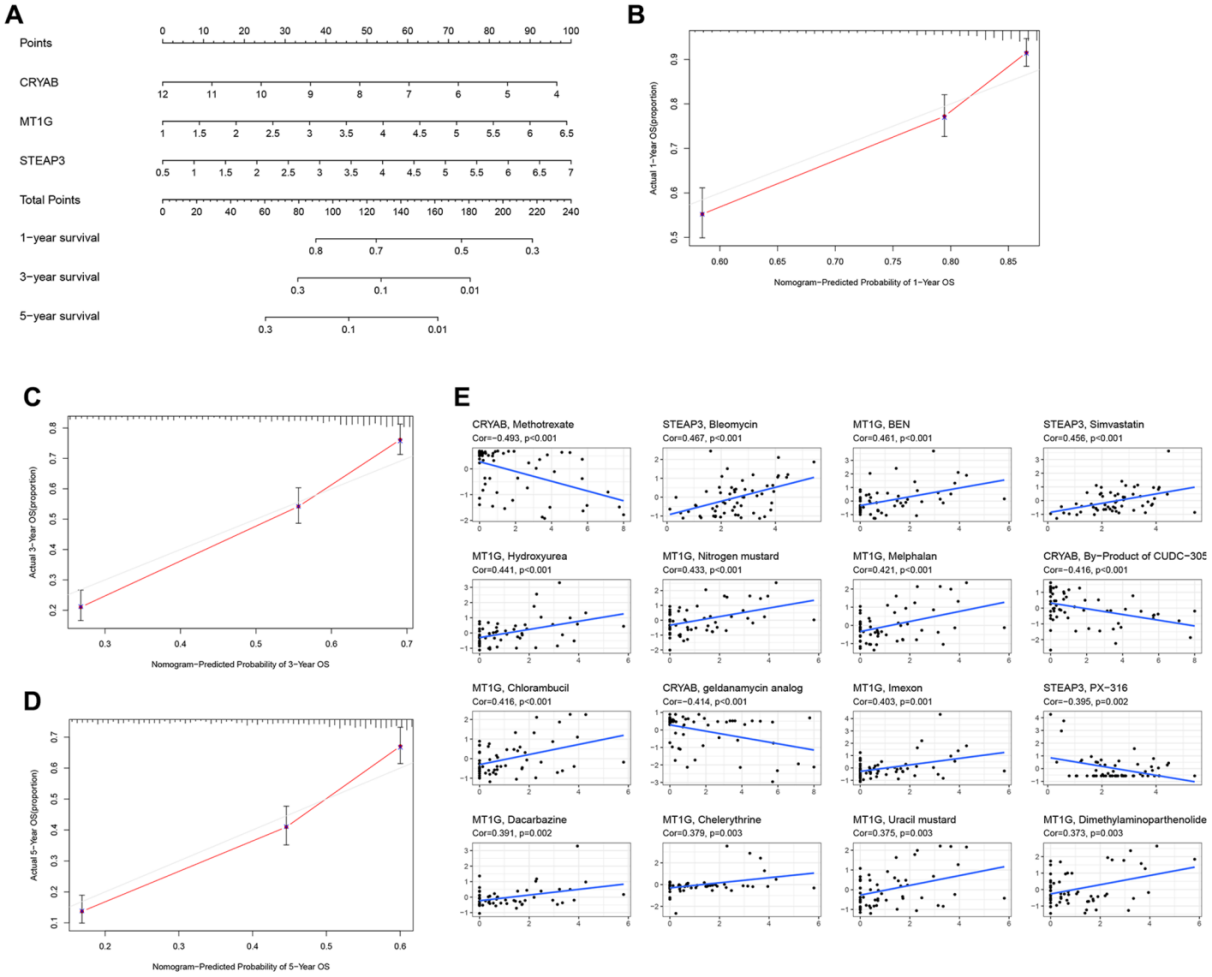
**Construction of a nomogram and drug relevance based on the 3 hub FRGs.** (**A**) Validation of the nomogram in the TCGA cohort. (**B**–**D**) Calibration maps used to predict the 1-year (**B**), 3-year (**C**), and 5-year survival (**D**). (**E**) The correlation between gene expression levels and drugs. The top 16 most relevant were visualized.

Briefly, the 3 FRGs were applied to design a nomogram (Figure 3A). The calibration curve results as presented in Figure 3B–3D exposed that the survival rate obtained by the model was nearly equal to the actual survival rate. Additionally, the relationship between level of the three FRGs and drugs was explored (Figure 3E). The drug data is obtained from the cellminer database (https://discover.nci.nih.gov/cellminer/loadDownload.do).

### Significant differences between low- and high-risk patients

**Figure 4:**
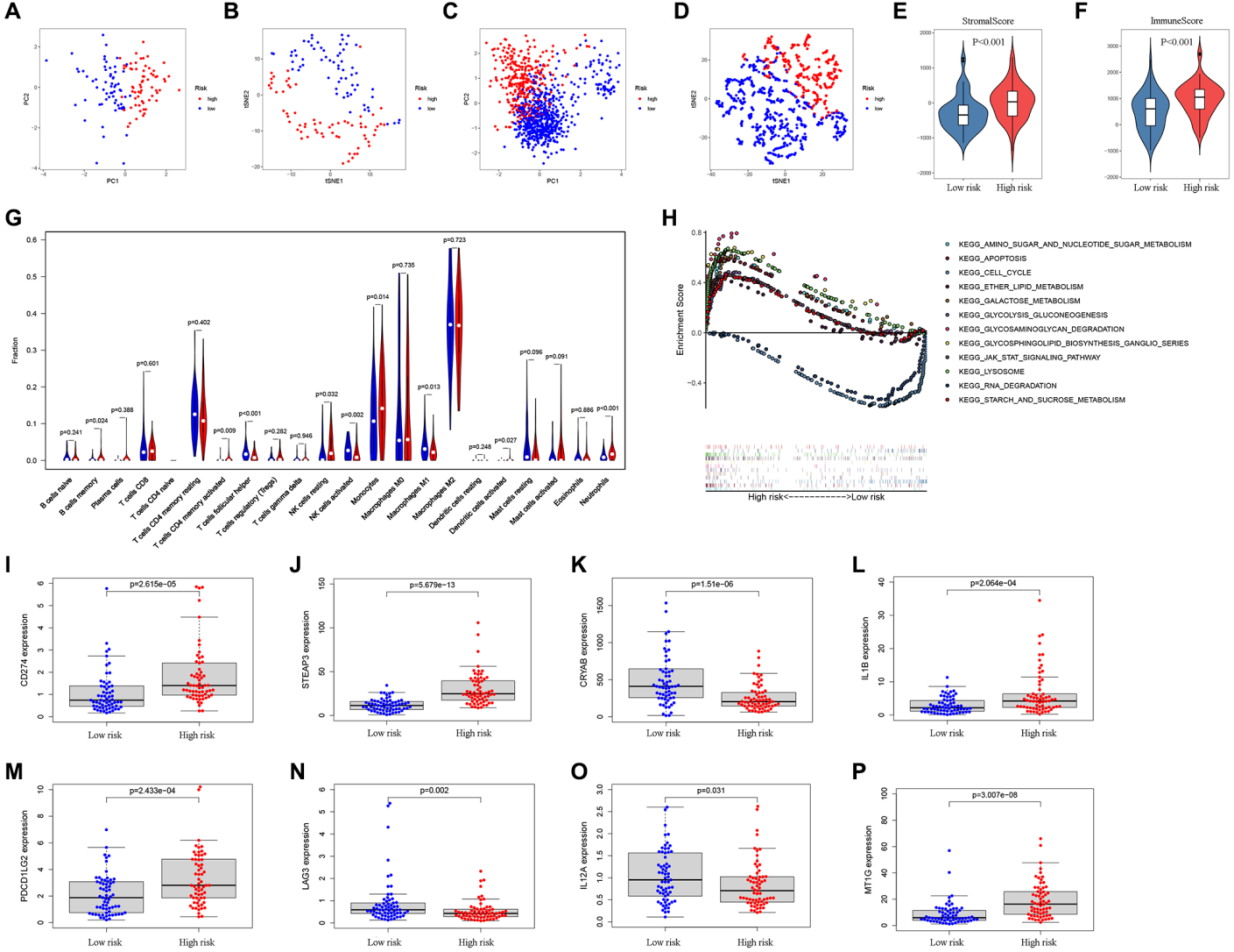
**Analysis of differences between high- and low-risk subgroups (tumor microenvironment, immune cell infiltration, and immune checkpoint regulators).** PCA (**A**) and t-SNE (**B**) analysis supported the stratification into two GBM subclasses the TCGA cohort. PCA (**C**) and t-SNE (**D**) analysis supported the stratification into two GBM subclasses the CGGA cohort. The comparison of stromal scores (**E**) and immune scores (**F**) in high- and low-risk subgroups. (**G**) The comparison of immune cell fractions between high- and low-risk subgroups. (**H**) The pathways enriched in high-risk GBM through GSEA analysis by enrichment map. (**I**–**P**) The key Immune checkpoint regulators with significant differential expression in the high- and low-risk subgroups.

Next, the patients were scored by the prognostic FRGs model, and grouped into low-risk and high-risk groups on the basis of median score. The results of PCA and t-SNE supported the classification of GBM patients into two subgroups by our FRGs signature (Figure 4A–4B). Additionally, verification in the CGGA cohort was also carried out and the outcomes are shown in Figure 4C–4D.

The ESTIMATE algorithm was adopted to assess the TCGA-GBM tumor microenvironment, and the results showed higher ImmuneScore and StromalScore in high-risk patients (Figure 4E–4F). The Supplementary Table 3 outlines the features of patients in both risk groups. The CIBERSORT algorithm analysis results found 21 immune cell types in TCGA-GBM samples (Figure 4G). Through GSEA enrichment analysis revealed that high-risk patient had enrichment in ferroptosis-related pathways, such as AMINO SUGAR AND NUCLEOTIDE SUGAR METABOLISM, APOPTOSIS, CELL CYCLE, ETHER LIPID METABOLISM, GALACTOSE METABOLISM, and GLYCOLYSIS GLUCONEOGENESIS (Figure 4H, Table 2).

**Table 2:** Gene set enrichment analysis in high-risk group.

**NAME**	**ES**	**NES**	**NOM *p*-val**	**FDR *q*-val**
KEGG_CELL_CYCLE	–0.590	–1.752	0.021	0.494
KEGG_RNA_DEGRADATION	–0.548	–1.601	0.036	0.433
KEGG_AMINO_SUGAR_AND_NUCLEOTIDE_SUGARMETABOLISM	0.614	1.812	0.005	0.052
KEGG_APOPTOSIS	0.593	1.994	0	0.011
KEGG_ETHER_LIPID_METABOLISM	0.448	1.463	0.040	0.187
KEGG_GLYCOLYSIS_GLUCONEOGENESIS	0.483	1.601	0.018	0.139
KEGG_GALACTOSE_METABOLISM	0.609	1.757	0.001	0.062
KEGG_GLYCOSAMINOGLYCAN_DEGRADATION	0.792	1.980	0	0.010
KEGG_GLYCOSPHINGOLIPID_BIOSYNTHESIS_GANGLIO_SERIES	0.676	1.707	0.009	0.087
KEGG_JAK_STAT_SIGNALING_PATHWAY	0.462	1.581	0.020	0.148
KEGG_LYSOSOME	0.671	2.050	0	0.011
KEGG_STARCH_AND_SUCROSE_METABOLISM	0.463	1.599	0.022	0.135

NOM *P* value < 0.01 was statistically significant. Abbreviations: FDR, False discovery rate.

In previous researches, immune checkpoint inhibitors e.g., PD-L1, PD-L2, and LAG3, were proposed as treatments for cancer. In the present study, CD274 (PD-L1), STEAP3, IL1B, PDCD1LG2 (PD-L2), and MT1G expressions were enriched in patients with high risk scores whereas the expression of CRYAB, LAG3, and IL12A was markedly enriched in those with low risk (Figure 4I–4P).

**Figure 5:**
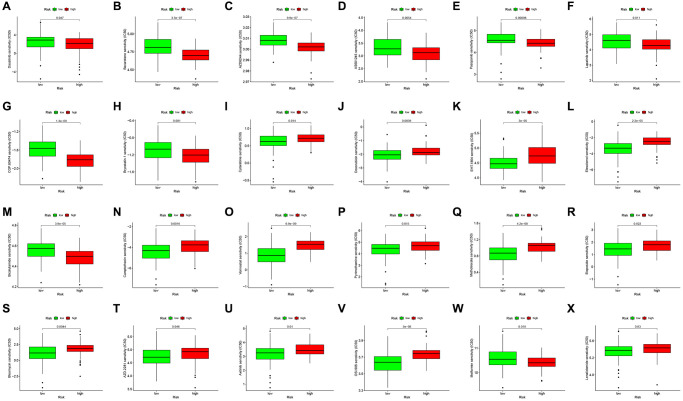
**Drug sensitivity analysis to drugs of high- and low-risk subgroups.** Differential chemotherapeutic responses in high- and low-risk patients (**A**–**X**).

To further study characterize drug responses in patients, the R package “pRRophetic” [35] was adopted to determine the half-maximal inhibitory concentration (IC50) of every GBM patient using the Genomics of Drug Sensitivity in Cancer (GDSC) website. Consequently, 24 drugs showed distinct estimated IC50 between low and high-risk GBM patients (Figure 5A–5X).

### Validation of the three genes in GBM cells

**Figure 6:**
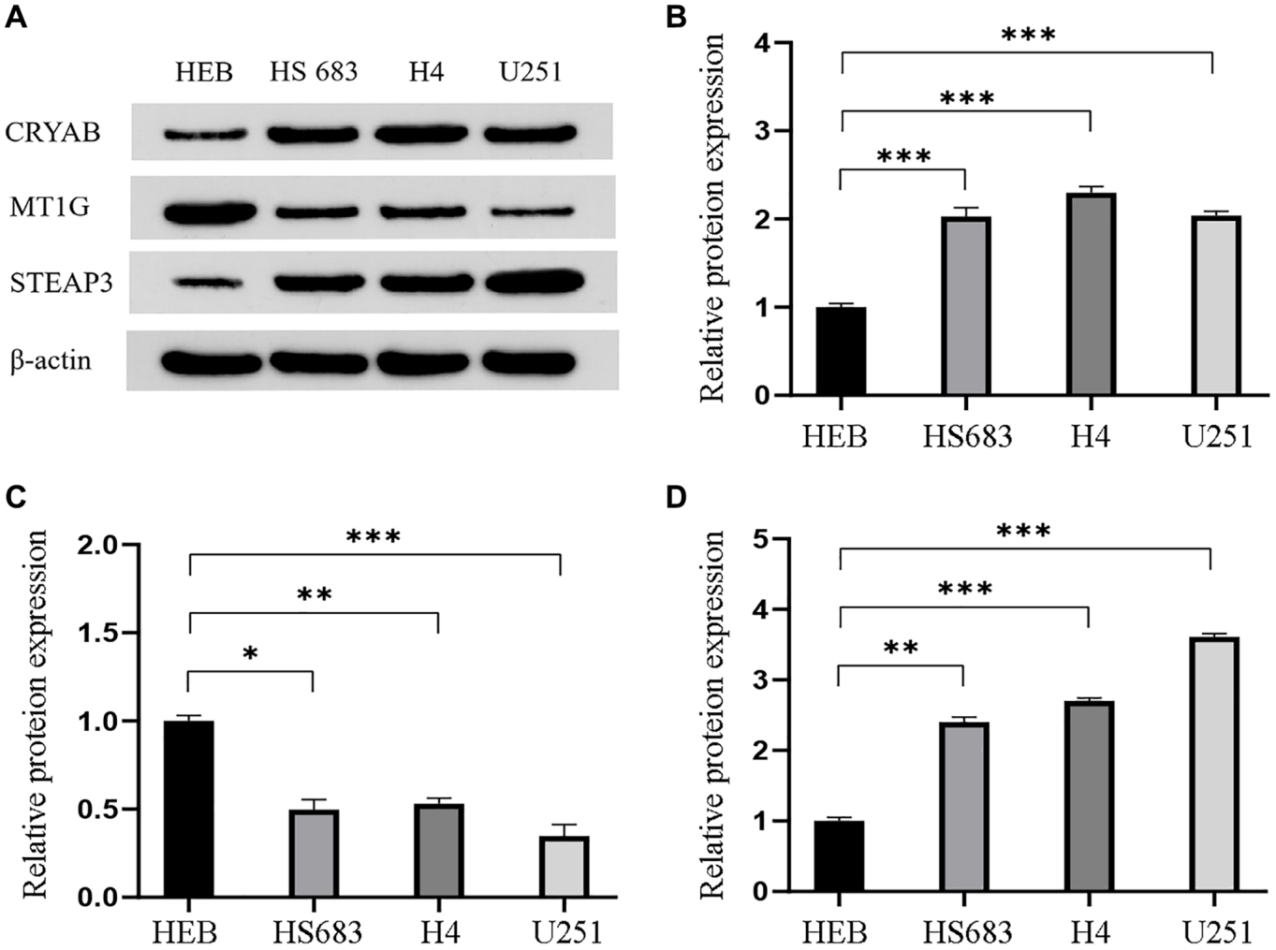
**Validation of the differential expression of the three genes in GBM cells.** (**A**) Western blot images and the relevant quantification (**B**–**D**) of CRYAB, MT1G, and STEAP3. Data are shown as mean ± SEM from three independent experiments, ^*^P < 0.05, ^**^P < 0.01, ^***^P < 0.001.

Western blot assay was conducted to quantify the protein level of the three genes in three GBM cell lines (HS 683 cells, H4 cells, and U251 cells), and in an HEB cell line (Figure 6A). Results presented in Figure 6 indicate that CRYAB (Figure 6B) and STEAP3 (Figure 6D) were upregulated in GBM cells than in HEB cell line, whereas MT1G (Figure 6C) was downregulated.

## DISCUSSION

GBM is one of the deadliest cancers with only a few approved therapies at present. Despite extensive studies on it over the last several decades, it remains incurable and fatal disease with little improvement in the survival rates for patients [36–39]. Thus, it boosted researchers to further explore on the mechanisms of GBM for novel prognostic biomarkers and therapeutic targets.

Ferroptosis is a form of cell death driven by iron dependent phospholipid peroxidation, which completely differs from other cell death such as necrosis, apoptosis, and autophagy for unique cell morphology, gene expression, and metabolic pathways. Cells undergoing ferroptosis gain a round shape before death with its distinctive feature, the smaller mitochondria, and increased mitochondrial membrane density. Previously, many pieces of researches have revealed that cancer cells in several cancers e.g., ovarian, lung, and breast cancers have high levels of iron relative to normal cells [40–45]. Moreover, it is reported that salinomycin can kill cancer stem cells by sequestering iron in lysosomes causing lysosomal membrane permeabilization and ferroptosis, suggesting that therapies targeting ferroptosis might be a possible strategy for cancer [46]. In another research, authors concluded that withaferin A can kill tumors in high-risk neuroblastoma via KEAP1 -Nrf2 axis, a noncanonical ferroptosis pathway [20]. Also, compelling evidence reported the lethality of GPX4 inhibitors in drug-resistant cells via ferroptosis, supporting that targeting GPX4 could act as a potential therapy to prevent drug resistance [47–49]. And in GBM, cisplatin co-delivered with small interference RNAs of GPX4 showed a significant superior therapeutic effect through a mechanism related to ferroptosis compared with cisplatin only *in vitro* and *in vivo* [21]. These evidences support the perspective applications of ferroptosis in GBM therapies. Nevertheless, the mechanisms of ferroptosis in GBM and its potential ability to prognosis are still not clear.

Therefore, we carried out current study to systematically evaluate the significance of FRGs in GBM with advanced computational tools. We assessed the expression profile of FRGs in GBM to get 32 DEGs. The relationship within these differentially expressed FRGs visualized by the PPI network suggesting TP53 with the most connected lines might play an important part in ferroptosis of cancer cells, while TP53 has already been one of the crucial dysregulated genes in most cancer types, indicating ferroptosis could also be one fundamental mechanism in GBM development. Next, we conducted GO and KEGG in the upregulated FRGs and downregulated FRGs. Results displayed that the upregulated FRGs are enriched in responses to oxidative stress except for ferroptosis itself. The close connection of FRGs and oxidative stress in GBM is readily comprehensible for the definition of ferroptosis cell death as an iron-dependent accumulation of lipid peroxidation. Some radical-trapping antioxidants can prevent ferroptosis. Meanwhile, the enrichment of the downregulated FRGs is associated with metabolism and biosynthesis as well as metabolism-related signaling pathways like PPAR and AMPK pathways. At present, metabolic changes are essential evidence for ferroptosis research. For example, iron abundance and lipid peroxidation level are two of the most critical indicators of ferroptosis. These findings revealed that ferroptosis, as well as FEGs, may have a significant impact on GBM development and give direction for in-depth studies on ferroptosis in GBM.

Based on the 32 differentially expressed FRGs, we observed 3 prognosis-associated candidate hub FRGs (CRYAB, MT1G, and STEAP3) as independent predictors to design a risk score model for prognostic prediction. The strong correlation between the low-risk subgroup of GBM and high survival rate suggested that those patients with low risk predicted by the FRGs signature are prone to a better prognosis with decent efficacy in both TCGA and CGGA cohorts. Furthermore, we performed various analyses to assess the correlation of FRGs signature and tumor microenvironment, which displayed higher ImmuneScore and StromalScore in the high-risk subgroup. More specifically, patients with high risk have a higher fraction of B cell memory (*p* = 0.024), T cell CD4 memory activated (*p* = 0.009), NK cells resting (*p* = 0.032), monocytes (*p* = 0.014), dendritic cells activated (*p* = 0.027), and neutrophils (*p* < 0.001), while those with low risk generate more T cells follicular helper (*p* < 0.001), and macrophages M1 (*p* = 0.013). For gene expressions of key immune checkpoints, there were significant differences in CD274, STEAP3, CRYAB, IL1B, PDCD1LG2, LAG3, IL12A, and MT1G in high- and low-risk subgroups. Our results uncovered that the FRGs signature may participate in tumor immunity and guide stratification and therapeutic strategies of immunotherapies in the future. In recent times, immunotherapies have made great progress in several solid tumors but could not improve the survival rate of GBM patients. Integrating immunotherapies with ferroptosis can provide a new insight to solve the challenges of current immunotherapies. Previous research found that during the anti-PD-L1 treatment, the dramatically elevated lipid peroxidation specific to ferroptosis along with inhibition of ferroptosis signaling cascades contributed thereby reducing tumor cells sensitivity. Above all, the functional mechanism of CRYAB, MT1G, and STEAP3 in ferroptosis and immunity should be further explored for potential treatments of GBM.

Thus, we analyzed the correlation between gene expression of CRYAB, MT1G, and STEAP3 and drugs, and also investigated the drug sensitivity in low- and high-risk subgroups, introducing possible drugs for certain subgroups divided by FRGs signature. Combination therapy with checkpoint inhibitors and drugs has proven to be a reliable therapy and is associated with a better prognosis [50–53].

At last, we preliminarily measured the protein expression of CRYAB, MT1G, and STEAP3 in GBM cell lines (HS 683, H4, and U251) compared with that in HEB cells. The rise of CRYAB and STEAP3 expression and fall of MT1G expression *in vitro* are consistent with their RNA level in TCGA sequencing data, supporting our bioinformatics analysis.

In summary, our novel FRGs-associated prognostic model for GBM could greatly provide access to the ferroptosis and pathogenesis in GBM and guide new prognostic biomarkers as well as therapeutic strategies for GBM.

## CONCLUSIONS

Overall, our study comprehensively analyzed the FRGs in GBM and builds an FRGs model (CRYAB, MT1G, and STEAP3) for prognosis and stratification of GBM patients. Subgroups with relative low or high-risk classified by the model have differences in ImmunoScore and StromalScore in tumor microenvironment and fraction of multiple immune cells, expression of immune checkpoint genes, and drug sensitivity. These findings can provide new insights for the development of new immunotherapy for GBM.

## Supplementary Materials

Supplementary Tables
