# Supplementary material for: Characterization of ferroptosis signature to evaluate the predict prognosis and immunotherapy in glioblastoma
Source: Aging (Albany NY). 2021 Jul 9;13(13):17655–72. doi: 10.18632/aging.203257 (PMC8312442; doi:10.18632/aging.203257)
Supplement: Supplementary Tables [file aging-13-203257-s001.pdf]

SUPPLEMENTARY TABLES

Supplementary Table 1. TCGA samples used in the study.

Sample ID (normal)	Sample ID (GBM)	Sample ID (GBM)	Sample ID (GBM)	Sample ID (GBM)	Sample ID (GBM)
TCGA-06–0675–11	TCGA-02–0047–01	TCGA-06–0646–01	TCGA-06–5856–01	TCGA-19–1389–02	TCGA-28–2513–01
TCGA-06–0678–11	TCGA-02–0055–01	TCGA-06–0649–01	TCGA-06–5858–01	TCGA-19–1390–01	TCGA-28–2514–01
TCGA-06–0680–11	TCGA-02–2483–01	TCGA-06–0686–01	TCGA-06–5859–01	TCGA-19–1787–01	TCGA-28–5204–01
TCGA-06–0681–11	TCGA-02–2485–01	TCGA-06–0743–01	TCGA-08–0386–01	TCGA-19–2619–01	TCGA-28–5207–01
TCGA-06-AABW-11	TCGA-02–2486–01	TCGA-06–0744–01	TCGA-12–0616–01	TCGA-19–2620–01	TCGA-28–5208–01
	TCGA-06–0125–01	TCGA-06–0745–01	TCGA-12–0618–01	TCGA-19–2624–01	TCGA-28–5209–01
	TCGA-06–0125–02	TCGA-06–0747–01	TCGA-12–0619–01	TCGA-19–2625–01	TCGA-28–5213–01
	TCGA-06–0129–01	TCGA-06–0749–01	TCGA-12–0821–01	TCGA-19–2629–01	TCGA-28–5215–01
	TCGA-06–0130–01	TCGA-06–0750–01	TCGA-12–1597–01	TCGA-19–4065–01	TCGA-28–5216–01
	TCGA-06–0132–01	TCGA-06–0878–01	TCGA-12–3650–01	TCGA-19–4065–02	TCGA-28–5218–01
	TCGA-06–0138–01	TCGA-06–0882–01	TCGA-12–3652–01	TCGA-19–5960–01	TCGA-28–5220–01
	TCGA-06–0141–01	TCGA-06–1804–01	TCGA-12–3653–01	TCGA-26–1442–01	TCGA-32–1970–01
	TCGA-06–0152–02	TCGA-06–2557–01	TCGA-12–5295–01	TCGA-26–5132–01	TCGA-32–1980–01
	TCGA-06–0156–01	TCGA-06–2558–01	TCGA-12–5299–01	TCGA-26–5133–01	TCGA-32–1982–01
	TCGA-06–0157–01	TCGA-06–2559–01	TCGA-14–0736–02	TCGA-26–5134–01	TCGA-32–2615–01
	TCGA-06–0158–01	TCGA-06–2561–01	TCGA-14–0781–01	TCGA-26–5135–01	TCGA-32–2616–01
	TCGA-06–0168–01	TCGA-06–2562–01	TCGA-14–0787–01	TCGA-26–5136–01	TCGA-32–2632–01
	TCGA-06–0171–02	TCGA-06–2563–01	TCGA-14–0789–01	TCGA-26–5139–01	TCGA-32–2634–01
	TCGA-06–0174–01	TCGA-06–2564–01	TCGA-14–0790–01	TCGA-27–1830–01	TCGA-32–2638–01
	TCGA-06–0178–01	TCGA-06–2565–01	TCGA-14–0817–01	TCGA-27–1831–01	TCGA-32–4213–01
	TCGA-06–0184–01	TCGA-06–2567–01	TCGA-14–0871–01	TCGA-27–1832–01	TCGA-32–5222–01
	TCGA-06–0187–01	TCGA-06–2569–01	TCGA-14–1034–01	TCGA-27–1834–01	TCGA-41–2571–01
	TCGA-06–0190–01	TCGA-06–2570–01	TCGA-14–1034–02	TCGA-27–1835–01	TCGA-41–2572–01
	TCGA-06–0190–02	TCGA-06–5408–01	TCGA-14–1402–02	TCGA-27–1837–01	TCGA-41–3915–01
	TCGA-06–0210–01	TCGA-06–5410–01	TCGA-14–1823–01	TCGA-27–2519–01	TCGA-41–4097–01
	TCGA-06–0210–02	TCGA-06–5411–01	TCGA-14–1825–01	TCGA-27–2521–01	TCGA-41-5651-01
	TCGA-06–0211–01	TCGA-06–5412–01	TCGA-14–1829–01	TCGA-27–2523–01	TCGA-76–4925–01
	TCGA-06–0211–02	TCGA-06–5413–01	TCGA-14–2554–01	TCGA-27–2524–01	TCGA-76–4926–01
	TCGA-06–0219–01	TCGA-06–5414–01	TCGA-15–0742–01	TCGA-27–2526–01	TCGA-76–4927–01
	TCGA-06–0221–02	TCGA-06–5415–01	TCGA-15–1444–01	TCGA-27–2528–01	TCGA-76–4928–01
	TCGA-06–0238–01	TCGA-06–5416–01	TCGA-16–0846–01	TCGA-28–1747–01	TCGA-76–4929–01

Supplementary Table 2. 60 Ferroptosis-related genes.

Ferroptosis-related genes
ACSL4, AKR1C1, AKR1C2, AKR1C3, ALOX15<br>ALOX5, ALOX12, ATP5MC3, CARS1, CBS<br>CD44, CHAC1, CISD1, CS, DPP4, FANCD2<br>GCLC, GCLM, GLS2, GPX4, GSS, HMGCR<br>HSPB1, CRYAB, LPCAT3, MT1G, NCOA4<br>PTGS2, RPL8, SAT1, SLC7A11, FDFT1, TFRC<br>TP53, EMC2, AIFM2, PHKG2, HSBP1, ACO1<br>FTH1, STEAP3, NFS1, ACSL3, ACACA, PEBP1<br>ZEB1, SQLE, FADS2, NFE2L2, KEAP1, NQO1<br>NOX1, ABCC1, SLC1A5, GOT1, G6PD, PGD<br>IREB2, HMOX1, ACSF2

Supplementary Table 3. Characteristics of patients in low and high-risk scores in TCGA cohort.

ID	futime	fustat	Age	Gender	Grade	Stage	T	M	N	Risk-group
TCGA-14–1829	218	0	57	MALE	unknow	unknow	unknow	unknow	unknow	Low risk
TCGA-19–5960	455	1	56	MALE	unknow	unknow	unknow	unknow	unknow	Low risk
TCGA-12–1597	675	1	62	FEMALE	unknow	unknow	unknow	unknow	unknow	Low risk
TCGA-32–4213	604	0	47	FEMALE	unknow	unknow	unknow	unknow	unknow	Low risk
TCGA-14–0871	880	1	74	FEMALE	unknow	unknow	unknow	unknow	unknow	Low risk
TCGA-14–0790	419	1	64	FEMALE	unknow	unknow	unknow	unknow	unknow	Low risk
TCGA-02–2483	466	0	43	MALE	unknow	unknow	unknow	unknow	unknow	Low risk
TCGA-06–2565	506	1	59	MALE	unknow	unknow	unknow	unknow	unknow	Low risk
TCGA-06–0221	603	1	31	MALE	unknow	unknow	unknow	unknow	unknow	Low risk
TCGA-28–5208	544	1	52	MALE	unknow	unknow	unknow	unknow	unknow	Low risk
TCGA-19–1787	385	1	48	MALE	unknow	unknow	unknow	unknow	unknow	Low risk
TCGA-26–5134	167	0	74	MALE	unknow	unknow	unknow	unknow	unknow	Low risk
TCGA-06–0174	98	1	54	MALE	unknow	unknow	unknow	unknow	unknow	Low risk
TCGA-32–5222	585	1	66	MALE	unknow	unknow	unknow	unknow	unknow	Low risk
TCGA-41–5651	460	1	59	FEMALE	unknow	unknow	unknow	unknow	unknow	Low risk
TCGA-06–0158	329	1	73	MALE	unknow	unknow	unknow	unknow	unknow	Low risk
TCGA-19–1390	772	1	63	FEMALE	unknow	unknow	unknow	unknow	unknow	Low risk
TCGA-12–0618	395	1	49	MALE	unknow	unknow	unknow	unknow	unknow	Low risk
TCGA-28–5209	442	0	66	FEMALE	unknow	unknow	unknow	unknow	unknow	Low risk
TCGA-32–1970	468	1	59	MALE	unknow	unknow	unknow	unknow	unknow	Low risk
TCGA-02–2485	470	0	53	MALE	unknow	unknow	unknow	unknow	unknow	Low risk
TCGA-06–5413	268	0	67	MALE	unknow	unknow	unknow	unknow	unknow	Low risk
TCGA-06–0744	1426	1	66	MALE	unknow	unknow	unknow	unknow	unknow	Low risk
TCGA-06–5411	254	1	51	MALE	unknow	unknow	unknow	unknow	unknow	Low risk
TCGA-06–0686	432	1	53	MALE	unknow	unknow	unknow	unknow	unknow	Low risk
TCGA-06–0129	1024	1	30	MALE	unknow	unknow	unknow	unknow	unknow	Low risk
TCGA-27–1837	427	1	36	MALE	unknow	unknow	unknow	unknow	unknow	Low risk
TCGA-32–2638	766	1	67	MALE	unknow	unknow	unknow	unknow	unknow	Low risk
TCGA-27–1834	1233	1	56	MALE	unknow	unknow	unknow	unknow	unknow	Low risk
TCGA-06–0219	22	1	67	MALE	unknow	unknow	unknow	unknow	unknow	Low risk
TCGA-28–5216	415	0	52	MALE	unknow	unknow	unknow	unknow	unknow	Low risk
TCGA-06–0130	394	1	54	MALE	unknow	unknow	unknow	unknow	unknow	Low risk
TCGA-27–2523	489	1	63	MALE	unknow	unknow	unknow	unknow	unknow	Low risk
TCGA-32–2634	693	0	82	MALE	unknow	unknow	unknow	unknow	unknow	Low risk
TCGA-26–5133	452	0	59	MALE	unknow	unknow	unknow	unknow	unknow	Low risk
TCGA-06–0743	803	1	69	MALE	unknow	unknow	unknow	unknow	unknow	Low risk
TCGA-06–0745	239	1	59	MALE	unknow	unknow	unknow	unknow	unknow	Low risk
TCGA-19–2629	737	1	60	MALE	unknow	unknow	unknow	unknow	unknow	Low risk
TCGA-27–1835	648	1	53	FEMALE	unknow	unknow	unknow	unknow	unknow	Low risk
TCGA-16–0846	119	1	85	MALE	unknow	unknow	unknow	unknow	unknow	Low risk
TCGA-06–2557	33	1	76	MALE	unknow	unknow	unknow	unknow	unknow	Low risk
TCGA-06–5417	155	0	45	FEMALE	unknow	unknow	unknow	unknow	unknow	Low risk
TCGA-06–5416	204	0	23	FEMALE	unknow	unknow	unknow	unknow	unknow	Low risk
TCGA-14–1825	232	1	70	MALE	unknow	unknow	unknow	unknow	unknow	Low risk
TCGA-28–1753	37	0	53	MALE	unknow	unknow	unknow	unknow	unknow	Low risk
TCGA-27–2526	87	1	79	FEMALE	unknow	unknow	unknow	unknow	unknow	Low risk

TCGA-06–0178	2681	1	38	MALE	unknow	unknow	unknow	unknow	unknow	Low risk
TCGA-06–2569	13	0	24	FEMALE	unknow	unknow	unknow	unknow	unknow	Low risk
TCGA-14–1402	975	1	58	FEMALE	unknow	unknow	unknow	unknow	unknow	Low risk
TCGA-06–0132	771	1	49	MALE	unknow	unknow	unknow	unknow	unknow	Low risk
TCGA-06–0157	97	1	63	FEMALE	unknow	unknow	unknow	unknow	unknow	Low risk
TCGA-06–2559	150	1	83	MALE	unknow	unknow	unknow	unknow	unknow	Low risk
TCGA-12–3652	1062	1	60	MALE	unknow	unknow	unknow	unknow	unknow	Low risk
TCGA-12–3650	333	1	46	MALE	unknow	unknow	unknow	unknow	unknow	Low risk
TCGA-28–5218	157	1	63	MALE	unknow	unknow	unknow	unknow	unknow	Low risk
TCGA-28–5215	335	1	62	FEMALE	unknow	unknow	unknow	unknow	unknow	Low risk
TCGA-28–1747	77	1	44	MALE	unknow	unknow	unknow	unknow	unknow	Low risk
TCGA-12–0616	448	1	36	FEMALE	unknow	unknow	unknow	unknow	unknow	Low risk
TCGA-06–0238	405	1	46	MALE	unknow	unknow	unknow	unknow	unknow	Low risk
TCGA-06–2564	181	0	50	MALE	unknow	unknow	unknow	unknow	unknow	Low risk
TCGA-19–2619	294	0	55	FEMALE	unknow	unknow	unknow	unknow	unknow	Low risk
TCGA-32–1982	142	1	76	FEMALE	unknow	unknow	unknow	unknow	unknow	Low risk
TCGA-26–1442	953	0	43	MALE	unknow	unknow	unknow	unknow	unknow	Low risk
TCGA-26–5132	286	0	74	MALE	unknow	unknow	unknow	unknow	unknow	Low risk
TCGA-41–2572	406	1	67	MALE	unknow	unknow	unknow	unknow	unknow	Low risk
TCGA-06–5418	83	1	75	FEMALE	unknow	unknow	unknow	unknow	unknow	High risk
TCGA-32–2616	224	1	48	FEMALE	unknow	unknow	unknow	unknow	unknow	High risk
TCGA-28–2509	145	0	77	FEMALE	unknow	unknow	unknow	unknow	unknow	High risk
TCGA-06–2558	380	1	75	FEMALE	unknow	unknow	unknow	unknow	unknow	High risk
TCGA-27–1831	505	1	66	MALE	unknow	unknow	unknow	unknow	unknow	High risk
TCGA-19–4065	214	0	36	MALE	unknow	unknow	unknow	unknow	unknow	High risk
TCGA-76–4928	94	1	85	FEMALE	unknow	unknow	unknow	unknow	unknow	High risk
TCGA-08–0386	548	1	74	MALE	unknow	unknow	unknow	unknow	unknow	High risk
TCGA-19–2620	148	1	70	MALE	unknow	unknow	unknow	unknow	unknow	High risk
TCGA-06–5412	138	1	78	FEMALE	unknow	unknow	unknow	unknow	unknow	High risk
TCGA-19–1389	141	1	51	MALE	unknow	unknow	unknow	unknow	unknow	High risk
TCGA-06–2561	537	1	53	FEMALE	unknow	unknow	unknow	unknow	unknow	High risk
TCGA-27–2524	231	1	56	MALE	unknow	unknow	unknow	unknow	unknow	High risk
TCGA-06-0138	737	1	43	MALE	unknow	unknow	unknow	unknow	unknow	High risk
TCGA-14–2554	532	1	52	FEMALE	unknow	unknow	unknow	unknow	unknow	High risk
TCGA-28–5204	454	1	72	MALE	unknow	unknow	unknow	unknow	unknow	High risk
TCGA-06–0646	175	1	60	MALE	unknow	unknow	unknow	unknow	unknow	High risk
TCGA-26–5136	577	1	78	FEMALE	unknow	unknow	unknow	unknow	unknow	High risk
TCGA-12–5295	454	1	60	FEMALE	unknow	unknow	unknow	unknow	unknow	High risk
TCGA-06–0139	362	1	40	MALE	unknow	unknow	unknow	unknow	unknow	High risk
TCGA-06–2567	133	1	65	MALE	unknow	unknow	unknow	unknow	unknow	High risk
TCGA-06–5408	357	1	54	FEMALE	unknow	unknow	unknow	unknow	unknow	High risk
TCGA-76–4927	535	1	58	MALE	unknow	unknow	unknow	unknow	unknow	High risk
TCGA-06–0878	218	0	74	MALE	unknow	unknow	unknow	unknow	unknow	High risk
TCGA-06–0644	384	1	71	MALE	unknow	unknow	unknow	unknow	unknow	High risk
TCGA-14–0789	342	1	54	MALE	unknow	unknow	unknow	unknow	unknow	High risk
TCGA-12–5299	98	1	56	FEMALE	unknow	unknow	unknow	unknow	unknow	High risk
TCGA-76–4929	111	1	76	FEMALE	unknow	unknow	unknow	unknow	unknow	High risk
TCGA-06–0210	225	1	72	FEMALE	unknow	unknow	unknow	unknow	unknow	High risk

TCGA-76–4931	279	1	70	FEMALE	unknow	unknow	unknow	unknow	unknow	High risk
TCGA-15–0742	419	1	65	MALE	unknow	unknow	unknow	unknow	unknow	High risk
TCGA-06–0211	360	1	47	MALE	unknow	unknow	unknow	unknow	unknow	High risk
TCGA-16–1045	883	1	49	FEMALE	unknow	unknow	unknow	unknow	unknow	High risk
TCGA-27–2519	550	1	48	MALE	unknow	unknow	unknow	unknow	unknow	High risk
TCGA-06–0750	28	1	43	MALE	unknow	unknow	unknow	unknow	unknow	High risk
TCGA-28–2513	222	0	69	FEMALE	unknow	unknow	unknow	unknow	unknow	High risk
TCGA-02–2486	618	1	64	MALE	unknow	unknow	unknow	unknow	unknow	High risk
TCGA-28–5207	343	1	71	MALE	unknow	unknow	unknow	unknow	unknow	High risk
TCGA-26–5139	48	0	65	FEMALE	unknow	unknow	unknow	unknow	unknow	High risk
TCGA-41–3915	360	1	48	MALE	unknow	unknow	unknow	unknow	unknow	High risk
TCGA-14–1034	485	1	60	FEMALE	unknow	unknow	unknow	unknow	unknow	High risk
TCGA-32–1980	36	1	72	MALE	unknow	unknow	unknow	unknow	unknow	High risk
TCGA-12–0821	323	1	62	MALE	unknow	unknow	unknow	unknow	unknow	High risk
TCGA-26–5135	270	1	72	FEMALE	unknow	unknow	unknow	unknow	unknow	High risk
TCGA-06–0649	64	1	73	FEMALE	unknow	unknow	unknow	unknow	unknow	High risk
TCGA-19–2625	124	1	76	FEMALE	unknow	unknow	unknow	unknow	unknow	High risk
TCGA-14–1823	543	1	58	FEMALE	unknow	unknow	unknow	unknow	unknow	High risk
TCGA-14–0817	164	1	69	FEMALE	unknow	unknow	unknow	unknow	unknow	High risk
TCGA-06–0125	1448	1	63	FEMALE	unknow	unknow	unknow	unknow	unknow	High risk
TCGA-12–3653	442	1	34	FEMALE	unknow	unknow	unknow	unknow	unknow	High risk
TCGA-06–5859	139	0	63	MALE	unknow	unknow	unknow	unknow	unknow	High risk
TCGA-41–2571	26	1	89	MALE	unknow	unknow	unknow	unknow	unknow	High risk
TCGA-06–5414	273	0	61	MALE	unknow	unknow	unknow	unknow	unknow	High risk
TCGA-12–0619	1062	1	60	MALE	unknow	unknow	unknow	unknow	unknow	High risk
TCGA-14–0736	460	1	49	MALE	unknow	unknow	unknow	unknow	unknow	High risk
TCGA-28–5220	388	1	67	MALE	unknow	unknow	unknow	unknow	unknow	High risk
TCGA-06–5858	187	0	45	FEMALE	unknow	unknow	unknow	unknow	unknow	High risk
TCGA-14–0787	68	1	69	MALE	unknow	unknow	unknow	unknow	unknow	High risk
TCGA-06–0152	375	1	68	MALE	unknow	unknow	unknow	unknow	unknow	High risk
TCGA-06–0184	2126	1	63	MALE	unknow	unknow	unknow	unknow	unknow	High risk
TCGA-06–0882	632	1	30	MALE	unknow	unknow	unknow	unknow	unknow	High risk
TCGA-06–0171	399	1	65	MALE	unknow	unknow	unknow	unknow	unknow	High risk
TCGA-41–4097	6	1	63	FEMALE	unknow	unknow	unknow	unknow	unknow	High risk
TCGA-06–0187	828	1	69	MALE	unknow	unknow	unknow	unknow	unknow	High risk
TCGA-27–1830	154	1	57	MALE	unknow	unknow	unknow	unknow	unknow	High risk
